# Weight management among school-aged children and adolescents: a quantitative assessment in a Ghanaian municipality

**DOI:** 10.1186/s12887-019-1772-4

**Published:** 2019-10-24

**Authors:** Daniel Gyamfi, Christian Obirikorang, Emmanuel Acheampong, Evans Adu Asamoah, Bernard Berko Sampong, Emmanuella Nsenbah Batu, Enoch Odame Anto

**Affiliations:** 1 Department of Medical Diagnostics, Faculty of Allied Health Sciences, Kwame Nkrumah University of Science and Technology, Kumasi, Ghana; 2 Department of Molecular Medicine, School of Medical Science, Kwame Nkrumah University of Science and Technology (KNUST), Kumasi, Ghana; 3 School of Medical and Health Sciences, Edith Cowan University, Joondalup, Western Australia Australia; 4 Department of Biochemistry, Dalian Medical University, Dalian, China

**Keywords:** Obesity, Overweight, Underweight, School-aged children, Adolescent

## Abstract

**Background:**

Childhood and adolescent overweight, obesity and underweight have become an issue of grave concern to both the developed and developing countries in context of global burden of non-communicable diseases. Unhealthy weight status is a significant public health issue for developing countries, of which Ghana is not excluded. This study evaluated the prevalence of overweight, obesity and underweight and its related factors among school-aged children and adolescents.

**Methods:**

A total of 1004 participants were randomly selected from six schools. A structured questionnaire on demography and socioeconomic status of students’ parents/guardians was completed by the selected students. Anthropometric parameters were measured, and body mass index (BMI) and waist-to-height ratio (WHtR) were calculated. BMI-for-age z-scores were used to categorize anthropometric data of the children as underweight, normal, overweight or obese. A cut-off value of > 0.50 was used to define obesity by WHtR.

**Results:**

Overweight prevalence of 13.8% and 12.6 was observed among basic school and high school students respectively based on BMI classification. Obesity prevalence of 8.8% was found in basic school students and 8.9% among high school students. Underweight was observed in 114 (11.3%) basic school students and 86 (8.6%) high school students. There was a difference in sex prevalence in unhealthy weight-behaviours; with more girls being overweight (19.4% vs 7.6%, *p* < 0.001) and obese (10.2% vs. 7.3%, *p* = 0.177) compared to boys. High WHtR found in 10.5% of basic students and 5.0% of high school students, with a statistical difference. Overweight/obesity was significantly associated with taking snacks before bed among basic school students [aOR = 10.45(5.95–18)] and high school students [aOR = 10.23(5.95–18.37)] respectively. Watching TV [aOR = 0.39(0.22–0.70)], sleeping during leisure periods [aOR = 0.43(0.23–0.81)] and bicycling as a means of transport [aOR = 0.37(0.19–0.72)] to school was protective of obesity among basic school students.

**Conclusion:**

High prevalence of unhealthy weight-related behaviours was observed among school-aged children in the Bekwai Municipality. Snacking before bed was a major factor promoting obesity among school-aged children while leisure behaviours such as TV watching, and sleeping were protective of obesity. Therefore, it is recommended to promote and support healthy eating habits among school-aged children which are likely beneficial in reducing the risk of childhood unhealthy weight-related behaviours.

## Background

Ghana’s economy is changing rapidly with an accelerated sustained economic growth which averages 8.0% per anum, and by the of 2019, Ghana’s growth target is expected to improve to 9.7% by industries, 7.3% by the agricultural sector and 6.2% as the financial sector continues to recover from its recent challenges [1]. These developmental promises are only seen in a certain part of the country, which widens the health disparities between the rich and the poor, and further burden an already overwhelmed health care system in the country. Moreover, the under-going economic transition changes the food environment facilitating rapid nutrition transition to an energy-dense, but nutrient-poor diet in conjunction with a sedentary lifestyle that could potentially affect the weight status of children and adolescents [2]. In addition, food security continues to be an issue with about 5% of the Ghanaian population being food insecure, hence undernutrition in rural communities [3, 4].

The World Health Organization in 2018 estimated that over 340 million children and adolescents (5–17 years) worldwide are either overweight or obese [5]. In Sub-Saharan Africa, the weighted average of school-aged children being overweight/obesity and obesity between the periods of 1960 and 2013 was reported to be 10.6 and 2.5% respectively [6]. In Ghana alone childhood overweight/obesity ranges between 7.6 and 17.4% where female sex stands the greater risk [7, 8]. Moreover, in the Northern parts of Ghana, the records of underweight school-aged children are reported to be as high as 29.8% according to the reports of Morgre et al. [8] These statistics indicate overweight/obesity transition among school-aged children and adolescents in Ghana and Africa as a whole. Strong links have also been shown between obesity and cardiovascular diseases. Subsequently, obese and overweight children are at high risk of developing hypertension, angina pectoris, type 2 diabetes mellitus and dyslipidemia [9]. Additionally, underweight has been associated with conditions such as osteoporosis; skin, hair and teeth problems; as well as cardiovascular outcomes.

Data on childhood and adolescent underweight and overweight/obesity in Sub-Saharan Africa including Ghana is limited especially childhood obesity since it is less recognized in developing countries [6, 8]. The 2014 annual composite reports in the Ashanti-Bekwai Municipality indicated that there was an increased incidence of non-communicable diseases (NCDs) mainly hypertension and diabetes mellitus from 1936 and 492 recorded in 2012 to 3618 and 1151 in 2014, respectively [10].

Despite positive advances made towards advertisements to prevent the intake of foods that is high in fats, sugars, and salt during television programmes aimed at children, limited action sits within the capabilities and responsibilities of health systems addressing such cases in the Bekwai municipality. Against this context, this study assessed the prevalence of underweight, overweight and obesity among school-aged within the Ashanti-Bekwai Municipality along with predisposing factors.

## Methods

### Study design/site

This was a cross-sectional study conducted among school-aged children and adolescents in 6 randomly selected schools within Ashanti-Bekwai Municipality. The Bekwai Municipal is one of the 30 administrative and political districts in the Ashanti Region of Ghana, located in the southern part of the region. It stretches over an area of 1937 km^2^ and lies within latitude 6° 00′N and 6° 30′ N and longitudes 1° 00 W and 1° 35 W. According the annual composite progress report of the Bekwai municipality, the estimated number of public and private school enrolment from primary up to senior high school in the year 2014 was 30,355 and 5540, respectively, featuring 191 schools [10]. The school attendance data for the population of Bekwai municipality is such that those attending basic and high school represent 76.7 and 23.3%, respectively, from an estimated population of 36,322 active schooling population out of the total enrolled students [11].

### Study population and selection of students

Using a multi-stage random sampling technique, students selected were within the age range of 5–17 years. To obtain a random distribution of the selected students from all classes, a proportionate random sample method was employed that included more students from larger classes.

### Sample size

To obtain a representative sample size for the entire student’s population (basic school and high school), at a confidence level of 95% and a margin of error of 0.036, the formula below was used:

$$ n=\frac{N}{1+N{(e)}^2} $$

Where *N* = total basic and high school student population, n = estimated sample size, e = margin of error.

Also, to ensure a relative proportion of the students by level, the total student population was stratified according to basic and high school using the formula:

$$ \mathrm{ni}=\mathrm{n}\frac{\mathrm{N}\mathrm{i}}{\mathrm{N}} $$

Where ni = sample size drawn from either primary or secondary schools, Ni = total population for either basic or high school, N = total student population, n = total estimated sample size. The sample size collected from both basic and high school were 702 and 302, respectively.

### Questionnaire administration

The selected students were asked to complete a structured questionnaire developed based on the review of related journals. It consisted of sections on demography (age, sex, parents’ occupation, and religion), eating habits (snacking status) and physical activity (means of transport and leisure activities). Parents or caretakers were made to answer the questionnaire for children below the age of 10 years. The ages of the selected students were determined using records available to the school. Children above 7 years were made to verbally confirm their age.

### Anthropometric measurements

Anthropometric measurements of weight, height, and waist of the selected students were taken. All measurements were done at the school premises. Height was measured to the nearest 0.1 cm using a portable height-rod stadiometer. The weights of the students were also measured using a weighing scale with the reading taken to the nearest 0.1 kg. Both measurements were taken on a flat surface with minimum clothing and without shoes. A good standing posture was maintained before each measurement was taken. A measurement of the hip (at the widest part of the buttocks) and waist (at the smaller circumference of the natural waist usually just above the belly button) of students were taken to the nearest centimetres using tape measures. Body mass index (BMI) and waist-to-height ratio (WHtR) was calculated as body weight divided by height squared (kg/m^2^) and the ratio of waist circumference (cm) to height (cm) respectively.

### Ethics approval and consent to participate

Ethics approval [CHRPE/AP/170/15] was sought from the Committee on Human Research and Publication Ethics (CHRPE) at the Kwame Nkrumah University of Science and Technology, School of Medical Sciences. Approval was also sought from the Bekwai Municipality Education Office in collaboration with the management of the six participating schools. Definite criteria and the process for deciding what individual research information was given to children and their families were agreed upon by the management. Written consent was obtained from each participant prior to entry into the study. Parental consent was sought from the participant below 16 years of age.

### Statistical analysis

Age and sex-specific prevalence of underweight, overweight and obesity were determined by body mass index for age percentiles using the criteria defined by the WHO cut-off points for school-aged children (WHO, 2014). According to the WHO cut-off points: overweight was ≥85th percentile; obesity, >95th percentile; underweight, <5th percentile; and normal, > 5th percentile to <85th percentile. Obesity by WHtR was defined according to WHtR cut-off values of > 0.5. Chi-square tests and cross-tabulations were used to compare and expressed categorical data as proportions (with Bonferroni correction for multiple comparisons. Logistic regression analysis adjusted for age was used to determine factors associated with underweight, overweight, obesity and higher WHtR prevalence with school as a fixed factor. All data were entered and managed in Microsoft Excel 2013 (Microsoft Corporation) and analysed using IBM Statistical Package for Social Scientists (SPSS) software version 22.0 (Chicago, USA). A level of *p* < 0.05 was considered statistically significant.

## Results

Female participants were the most presented from both basic and high schools (52.8 and 53.3%) respectively. Majority of high school participants and basic schools’ students were within the age bracket 10–15 years (71.2% vs 64.1%). A higher percentage of both basic and high school students had parents with skilled occupations but differed significantly in terms of proportions (58.5% vs 69.5%, *p* = 0.001). A considerable proportion of the students were from Christian homes in both basic and high school categories (77.8% vs. 73.2%). The commonest means of transport used by students from both basic and high school was by car (60.5% vs. 55.3%). Watching television (TV) was the most activity performed by participants during their leisure periods (32.5 and 38.4%, respectively) for basic and high school. More than half of students from both basic and high school participants took snacks before breakfast (58.7% vs 55.3) respectively Between lunch and supper snacking status was high among participants from high school (27.5%) than basic school (20.4%) (Table 1).

**Table 1:** General characteristics of the studied population stratified by school context

Variables	School environment		*P*-value
	Basic School (*n* = 702) n (%)	High School (*n* = 302) n (%)	
Sex^a^			0.945
Male	331 (47.2)	141 (46.7)	
Female	371 (52.8)	161 (53.3)	
Age (years) ^b^			< 0.0001
5 < x < 10	218 (31.1)	0	
10–15	450 (64.1)	215 (71.2)	
> 15	34 (4.8)	87 (28.8)	
Occupation ^b^			0.001
Unskilled	291 (41.5)	92 (30.5)	
Skilled	411 (58.5)	210 (69.5)	
Religion			0.116
Christian	546 (77.8)	221 (73.2)	
Islam	156 (22.2)	81 (26.8)	
Means of transport ^b^			0.209
Bicycle	120 (17.1)	65 (21.5)	
Car	425 (60.5)	168 (55.6)	
Trekking	157 (22.4)	69 (22.8)	
Leisure behaviours ^b^			0.221
Football	151 (21.5)	52 (17.2)	
Reading	95 (13.5)	45 (14.9)	
TV watching	228 (32.5)	116 (38.4)	
Sleeping	147 (20.9)	62 (20.5)	
Others	81 (11.5)	27 (8.9)	
Snacking Status ^b^			0.039
Before Bed	147 (20.9)	52 (17.2)	
Before breakfast	412 (58.7)	167 (55.3)	
Btn lunch and supper	143 (20.4)	83 (27.5)	

All values are presented as frequency and subset of school category proportions

^a^Fischer exact test

^b^ Chi-Square test, *P* < 0.05 is considered stattistically significant

**Fig. 1:**
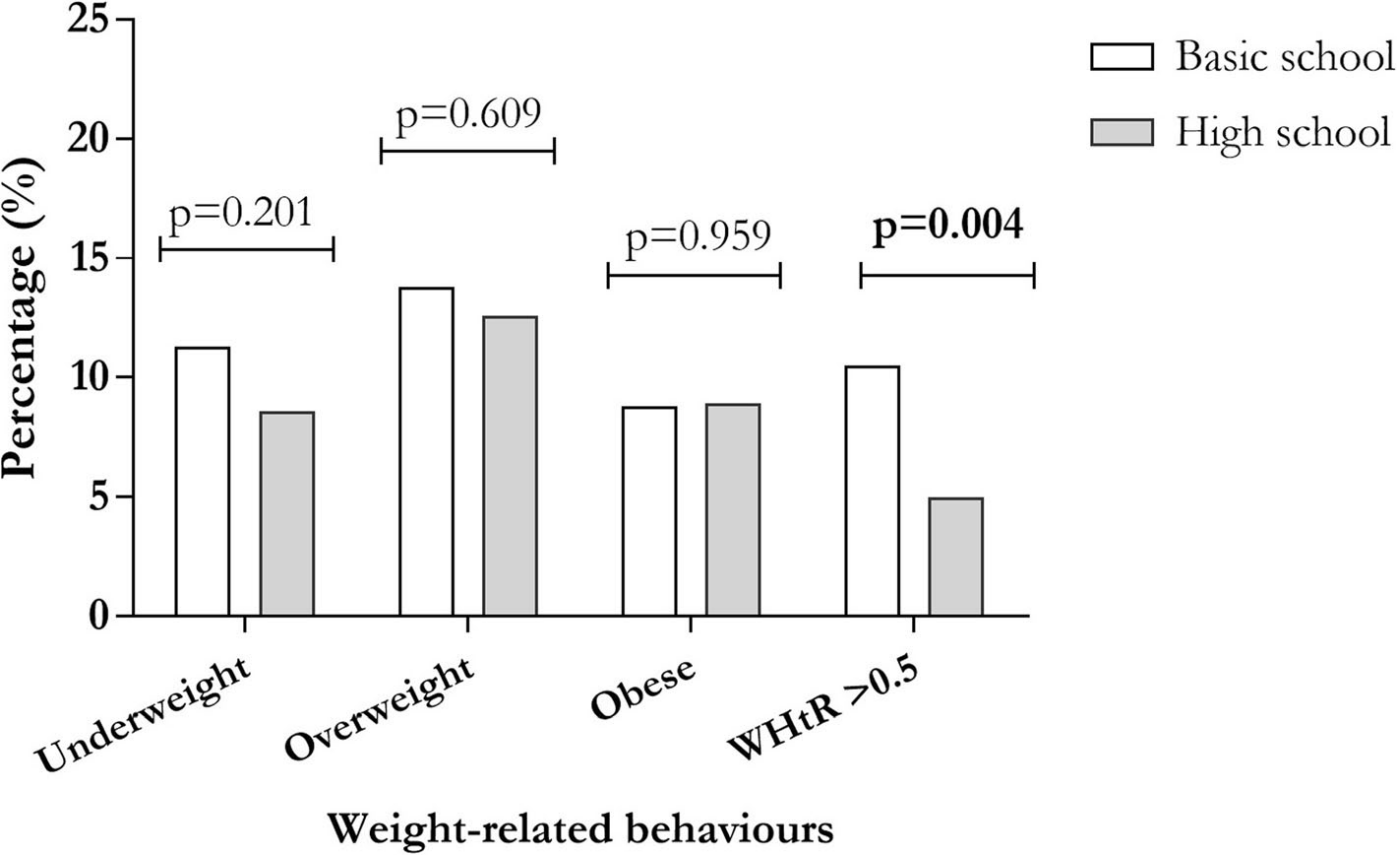
Prevalence of unhealthy weight-related behaviours by school context

As shown in Fig. 1, underweight based BMI classification was found in 11.3% of basic school students and 8.6% high school students with no significant difference (*p* = 0.201). Overweight defined by BMI classification was higher among basic school students compared to high school students (13.8% vs. 12.6%, *p* = 0.608). Obesity identified based on BMI classification was found in equivalent proportions of basic and high schools’ students (8.8% vs. 8.9, *p* = 0.959).

Using a cut-off of > 0.5, higher WHtR was significantly observed in basic school students compared to high school students (10.5% vs. 5.0% *p* = 0.004). The prevalence of underweight by BMI classification was significantly higher among basic schoolboys compared to girls (15.4% vs 7.5%, *p*-value =0.001). Also, underweight was prevalent in 8.5 and 8.7% of boys and girls respectively among high school students. The prevalence of overweight by BMI classification was significantly higher among basic schoolgirls compared to boys (19.4% vs 7.6%, *p*-value < 0.0001). Overweight (19.4% vs 7.6%) and obesity (10.2% vs 7.3%) were prevalent among basic school females compared to males. However, among high school students, overweight (13.5% vs 11.8%) and obesity (9.2% vs 8.7%) were more prevalent among males compared to females. Higher WHtR was prevalent in higher proportion among basic school boys (16.0%) compared to basic school girls (5.7%) (Table 2).

**Table 2:** BMI and WHtR status of the study population stratified by school context

Weight-related behaviours	Basic school			Secondary school		
	Boys (*n* = 331)	Girls (*n* = 371)	*P*-value	Boys (*n* = 141)	Girls (*n* = 161)	*P*-value
Underweight (%)	15.4%	7.5%	0.001	8.5%	8.7%	0.951
Healthy weight (%)	69.8%	62.8%	0.051	68.8%	70.8%)	0.665
Overweight (%)	7.6%	19.4%	< 0.0001	13.5%	11.8%	0.657
Obese (%)	7.3%	10.2%	0.177	9.2%	8.7%	0.879
WHtR > 0.5 (%)	16.0%	5.7%	< 0.0001	2.8%	6.8%	0.110

All values are presented as and compared using Chi-square test of trend, *P* < 0.05 is considered stattistically significant

The proportion of underweight prevalent among students within the 5 < x < 10 age range was higher compared to students within the 10–15 years and > 15 years age bracket (18.3% vs 9.0% vs 5.8%, *p* < 0.0001). Also, higher WHtR was more prevalent among students within the age range 5 < x < 10 compared to students within the > 15 years age bracket (12.4% vs 4.8%). The prevalence of underweight was significantly higher among Muslims students compared to Christian students (15.2% vs 9.0%, *p* = 0.006). Statistically significance difference was observed for overweight and obesity categories according to students’ with higher prevalence observed among students that take snacks before bed (42.7 and 23.1%) compared to students that takes snacks before breakfast (4.7 and 7.9%) and between lunch and supper (7.1 and 7.1%) (Table 3).

**Table 3:** Prevalence of unhealthy weight-related behaviours by socio-demographic characteristics

Variable	Underweight (%)	Overweight (%)	Obesity (%)	WHtR (> 0.5) (%)
Age (years)				
5 < x < 10	18.3	8.7	9.6	12.4
10–15	9.0	13.8	7.7	7.6
> 15	5.8	15.9	13.0	4.8
*P*-value	< 0.0001	0.070	0.052	0.012
Occupation				
Unskilled	10.2	13.6	9.4	7.3
Skilled	10.6	13.4	8.5	9.8
*P*-value	0.841	0.928	0.621	0.212
Religion				
Christian	9.0	13.8	8.9	9.3
Islam	15.2	12.2	8.9	7.6
*P*-value	0.006	0.528	1.000	0.422
Means of transport				
Bicycle	13.5	9.2	5.4	11.1
Car	9.9	14.3	9.3	7.8
Trekking	9.3	14.6	10.6	9.7
*P*-value	0.311	0.170	0.213	0.283
Leisure behaviours				
Football	11.8	15.3	7.4	14.3
Reading	12.1	15.7	9.3	10.7
TV watching	11.0	12.5	6.4	4.7
Sleeping	8.6	11.0	9.6	8.6
Others	7.4	14.8	17.6	10.2
*P*-value	0.594	0.608	0.009	0.003
Snacking Status				
Before Bed	10.1	42.7	23.1	13.6
Before breakfast	8.5	5.2	4.7	7.9
Between lunch and supper	15.9	8.8	7.1	7.1
*P*-value	0.017	< 0.0001	< 0.0001	0.029

Logistic regression analysis adjusted for age demonstrated that female sex was significantly associated with increased likelihood of overweight/obesity (aOR = 2.16) but decreased the likelihood of being underweight (aOR = 0.61) among basic school students. Taking snacks before bed was significantly associated with 10.45- and 10.23-times likelihood of obesity/underweight among basic school and high school students respectively. Football playing (aOR = 0.47), TV watching (aOR = 0.39) and sleeping (aOR = 0.43) during leisure periods were associated with a decreased likelihood of overweight/obesity among basic school-aged students. Moreover, basic school students that traveled by means of bicycling was associated with less likelihood to be overweight/obese (aOR = 0.37) but increased odds of being lean (aOR = 2.18). In addition, taking snacks before breakfast was significantly associated with less likelihood of underweight (aOR = 0.39, *p* = 0.001) and overweight/obese (aOR = 0.54) among basic school students (Table 4).

**Table 4:** Logistic regression analysis of factors associated with underweight, overweight and obesity among the students

Variable	Basic School		High School	
	Underweight aOR (95% CI)	Overweight/Obesity aOR (95% CI)	Underweight aOR (95% CI)	Overweight/Obesity aOR (95% CI)
Sex				
Female	0.61 (0.37–1.00) ^a^	2.34 (1.60–3.43) ^b^	0.94 (0.41–2.15)	0.92 (0.0.53–1.61)
Male	1	1	1	1
Religion				
Christian	0.54 (0.32–0.92) ^a^	0.95 (0.62–1.15)	0.59 (0.25–1.39)	1.41 (0.73–2.72)
Muslim	1	1	1	1
Occupation				
Unskilled	0.82 (0.50–1.38)	1.05 (0.73–1.50)	1.86 (0.81–4.30)	0.96 (0.52–1.75)
Skilled	1	1	1	1
Means				
Bicycle	2.18 (1.01–4.71) ^a^	0.37 (0.19–0.72) ^b^	0.61 (0.18–2.01)	0.72 (0.31–1.64)
Car	1.54 (0.79–3.00)	0.92 (0.60–1.40)	0.58 (0.23–1.15)	0.82 (0.42–1.60)
Trekking	1	1	1	1
Leisure behaviours				
Football	2.24 (0.87–6.41)	0.47 (0.26–0.87) ^a^	0.82 (0.13–5.45)	0.84 (0.29–2.48)
Reading	2.24 (0.82–7.15)	0.63 (0.33–1.21)	1.46 (0.26–8.34)	0.73 (0.24–2.28)
TV watching	1.69 (0.64–4.42)	0.39 (0.22–0.70) ^b^	1.41 (0.29–6.88)	0.62 (0.23–1.67)
Sleeping	1.27 (0.45–3.56)	0.43 (0.23–0.81) ^b^	0.83 (0.14–4.95)	0.84 (0.29–2.40)
Other activities	1	1	1	1
Snacking				
Before Bed	1.55 (0.73–3.30)	10.45 (5.95–18.37) ^b^	1.72 (0.46–6.40)	10.23 (4.42–23.68) ^b^
Before Breakfast	0.33 (0.19–0.57) ^b^	0.54 (0.31–0.94) ^a^	0.67 (0.27–1.65)	0.66 (0.30–1.41)
BLS	1	1	1	1

All values are presented as adjusted odds ratio (aOR) (95% confidence interval), BLS-between lunch and supper, CI-confidence interval

^a^denotes significant level at 0.05 α-level

^b^denotes significant at 0.01 α-level

Upon logistic regression analysis, female sex was associated with decreased odds of higher WHtR (aOR = 0.32, *p* < 0.0001) among basic school children. Moreover, basic school students with unskilled parents were associated with less likelihood of higher WHtR (aOR = 0.57, *p* = 0.036) (Table 5).

**Table 5:** Logistic regression analysis of factors associated with high WHtR (> 0.5) among study participants

	Basic school		High Scholl	
Variable	aOR (95% CI)	*P*-value	aOR (95% CI)	*P*-value
Sex				
Female	0.32 (0.19–0.54)	< 0.0001	2.43 (0.75–7.85)	0.137
Male	1			
Religion				
Christian	1.40 (0.75–2.63)	0.292	0.73 (0.24–2.22)	0.582
Muslim	1		1	
Occupation				
Unskilled	0.57 (0.34–0.97)	0.036	1.64 (0.56–4.79)	0.364
Skilled	1		1	
Means of Transport				
Bicycle	1.91 (0.95–3.86)	0.070	0.0	–
Car	0.86 (0.46–1.60)	0.639	0.59 (0.20–1.17)	0.329
Trekking	1		1	
Leisure Activity				
Football	1.77 (0.81–3.89)	0.154	0.00	–
Reading	0.79 (0.30–2.06)	0.623	3.97 (0.45–34.95)	0.214
TV watching	0.41 (0.17–1.01)	0.052	0.95 (0.10–8.86)	0.962
Sleeping	0.77 (0.32–1.82)	0.551	1.79 (0.19–16.80)	0.611
Other activities	1		1	
Snacking Status				
Before Bed	1.72 (0.85–3.49)	0.135	3.36 (0.59–19.06)	0.171
Before Breakfast	0.90 (0.47–2.18)	0.738	2.36 (0.50–11.20)	0.280
Before lunch and supper	1		1	

All values are presented as adjusted odds ratio (aOR) (95% confidence interval), CI-confidence interval, *P *< 0.05 is considered stattistically significant

## Discussion

The results of this study indicated an overweight prevalence of 13.8 and 12.6%, among basic school and high school students respectively. Also, an obesity prevalence of 8.8% among basic school students and 8.9% among high school students were observed. Additionally, obesity defined by WHtR > 0.05 was prevalent in 10.5% of basic school students and 5.0% among high school students. Eating snacks before breakfast and being female accounted for a significant prevalence of overweight/obesity among school-aged children. Hitherto, watching TV, sleeping, and football playing as leisure activities as well as bicycling as a means of transport to school was protective of overweight/obesity among basic school-aged children. Moreover, a considerable prevalence of underweight (11.3% among basic school students and 8.6% among high school students) was also observed, which was an indication of the co-existence of undernutrition and overnutrition, facilitating the transition of unhealthy weight-related behaviours in school-aged children within the Bekwai Municipality.

Overweight and obesity principally reflect positive energy balance; physical inactivity and poor dietary habits are two key modifiable factors that Overweight and obesity principally reflect positive energy balance; physical inactivity and poor dietary habits are two key modifiable factors that can influence this balance in a population. Aryeetey et al. [12], in a cross-sectional study conducted among school-aged children in urban Ghana, reported an overweight/obesity prevalence of 15% and indicated that low physical activity participation was associated with overweight and obesity among school-aged children which is partly consistent with findings in this present study. A similar cross-sectional study conducted in the Tamale Metropolis in Ghana, reported prevalence rates of 9.8 and 7.5% for overweight and obesity and indicated that obesity/overweight prevalence is associated with attending a private school, high level of parental education, playing computer/video games and eating food at the school canteen [13]. In the same year, [8] another study among school-aged children between 5 and 14 years age group in the Tamale metropolis reported an overweight/obesity prevalence of 17.4% which is in concordance to our present finding. We should underscore that childhood obesity is prominent in the Ghanaian population, therefore, the contextualization of obesity prevalence in Ghana requires a combination of factors such as dietary and physical activities that have a concurrent effect on obesity prevalence. Evidence from the literature indicates that increasing weight is the result of caloric imbalance and this is mediated by genetic, behavioral, and environmental factors driving the pathogenesis [14].

The evidence of childhood obesity as an emerging public health-related problem in the Bekwai Municipality is imminent [9]. Interestingly, the annual composite reports of the Bekwai municipality indicated that obesity prevalence among school-aged children was less than 1% [10]. Thus, the combined prevalence of overweight and obesity demonstrated in this present study is indeed indicative of increased future trends of childhood obesity coupled with attendant problems within the Municipality. In recent systematic review and meta-analysis by Muthuri et al. [6] reported a weighted average of overweight/obesity and obesity only to be 10.6 and 2.5% respectively, from 1960 to 2013 in studies reviewed from the entire Sub-Saharan African Zone. The results of our study are consistent with recent reports from several studies elsewhere in Africa and this highlights that overweight/obesity transition is evident among school-aged children in Sub Saharan Africa [7, 8, 15, 16].

Our findings showed a positive relationship between female sex and overweight /obesity, among basic school students but not high school students, which is consistent with previous findings [6–8]. A cross-sectional study conducted by Toriola et al. [17], reported no significant sex differences for BMI between boys and girls up to 13 years. However, the same study reported that increased age (> 13 years) was associated with significant sex differences for BMI.

In this study, basic school students represented a group whose peak ages range from 5 to 15 years compared to high school students who were older (10 to greater than 15 years). The records of sex-specific obesity in our findings indicate that overweight and obesity were prevalent among 19.4 and 10.2% respectively among basic school-aged girls, compared to boys whose prevalence was 7.6 and 7.3% respectively. Comparatively, more boys were overweight and obese than girls in high schools. To some extent, sex dissimilarities with respect to the behavioral elements of overweight/obesity; calorie intake and physical activity, has been reported. The evidence existing presents findings in one aspect, that girls give more attention to healthy eating behaviours to meet nutritional recommendations than boys [18], whiles others also report that boys engage in vigorous-intensity physical activities than girls [8]. Thus, sex-specific unhealthy weight-related behaviours are more specific to the study setting and existing practices of the study subjects.

From the discrete point view, childhood obesity is the result of an imbalance between the calories a child consumes as food and beverages and the calories a child uses to support normal growth and development, metabolism, and physical activity [19]. The disproportion between calories consumed and calories used can result from the influences and interactions of a number of factors, comprising genetic, behavioral, and environmental factors [14]. It has, therefore, become imperative that the interactions among these factors rather than a single factor should be critically considered in tackling the cause of underweight and obesity/overweight. Several studies have also reported that childhood overweight/obesity is negatively associated with physical activity and positively associated with sedentary activities [20].

We found that football playing during leisure periods and bicycling as a means of transport to school, which represents vigorous-intensity exercise, was protective of overweight/obesity. Also, snacking status, especially, taking snacks before bed was associated with a significant prevalence of overweight (42.7%) and obesity (23.1%) among school-aged students. In a review report by Nuru and Mamang [21], children who eat a lot of snacks more frequently stand a higher risk of obesity, compared to those who eat snack without hungry feel. In a more recent study, Nisak et al., [22] reported that energy intake from snacking was found to be higher in obese children, which was attributed to the highest frequency of energy-dense food intakes such as flavored drinks and desserts. Eating a quick and nutritious snack just before bed can have nutritional benefits for school-aged students. However, if the resorts are flavored drinks and deserts, then the child has a higher risk of obesity. Although we did not assess the nutrition status of snacks in this study, the content of foods consumed by children before bed might explain the high prevalence rate of obesity with respect to snacking before bed.

Although watching TV and sleeping is a proxy measure for sedentary behaviours, we observed that TV watching and sleeping among basic school children during leisure periods was protective of obesity. Leisure periods denote times of quality experience or free time spent from basic routine activities. Several studies have reported, that sedentary leisure such as TV watching and sleeping is associated with high BMI levels among children and adolescents [23, 24], which is inconsistent with our findings. However, findings of Zimmerman and Bell [25], indicated that the content of TV shows is the major underlying factor for obesity prevalence among children. Thus, children that alternatively watch educational programs were not at risk of obesity compared to children who watched commercial programs. Zimmerman and Bell reported that most commercial programs are accompanied by junk food and sugar-sweetened beverages advertisement (similar to most commercial TV shows in Ghana) which influences the eating pattern of children, thus enhancing obesity prevalence. We, therefore, presumed that the content of TV watching rather than the practices among basic school children influenced the protective behaviour of TV watching for obesity in our study. Moreover, duration and timing of sleeping in alternating ways influence the risk of overweight or obesity during childhood [26]. Poor or lack of sleep among children has been reported to disrupt appetite-regulating hormones, thus causing weight gain [27, 28]. Thus, these reasons could affirm the protective role of sleeping for obesity prevalence among basic school children, found in our study.

Even though reports of this present study are comparable to other literature, the study design used in this present study was a cross-sectional which was not effective to establish a causal effect relationship between snacking status; leisure time activities; and childhood overweight/obesity. Moreover, the content of TV programs the children watched as well as the kind of food eating for snacks before bed was not assessed to effectively elaborate the effects on weight-related behaviours. It is, therefore, recommended that more elaborate research into the objective measurement of these exposures should be carried out to establish their true relationship with childhood overweight/obesity in the Ghanaian context. Again, constant monitoring of the nutritional status in school children should be considered by considering interventions such as breakfast and lunch clubs. Also, more physical education lessons should be included in the basic and high school curriculum.

## Conclusion

A high prevalence of childhood overweight, obesity and underweight were found among school-aged children and adolescents. Snaking independently and significantly promotes obesity, overweight and underweight prevalence among school-aged children and adolescents whereas leisure behaviours such as TV watching, and sleeping were protective of obesity. Promoting and supporting healthy eating habits in this population is likely beneficial in reducing the risk of childhood unhealthy weight-related behaviours.

## Data Availability

The datasets used and analyzed during the study are available from the corresponding author on reasonable request.

## Abbreviations

BMI: Body mass index
WHtR: Waist-to-height Ratio
